# THADA inhibits autophagy and increases 5-FU sensitivity in gastric cancer cells via the PI3K/AKT/mTOR signaling pathway

**DOI:** 10.22038/IJBMS.2023.72055.15668

**Published:** 2024

**Authors:** Xianke Lin, Jiajia Pan, Hammza Hamoudi, Jiren Yu

**Affiliations:** 1 Department of Gastrointestinal Surgery, The First Affiliated Hospital, Zhejiang University School of Medicine, Hangzhou, China; 2 Department of Hematology, The First Affiliated Hospital, Zhejiang University College of Medicine, Hangzhou, China; 3 Zhejiang University College of Medicine, Hangzhou, China; # These authors contributed equally to this work

**Keywords:** Autophagy, Fluorouracil, Human, Stomach Neoplasms, THADA protein, TOR Serine-Threonine- Kinases

## Abstract

**Objective(s):**

5-Fluorouracil (5-FU) is currently the main drug used in chemotherapy for gastric cancer (GC). The main clinical problems of 5-FU therapy are insensitivity and acquired resistance to 5-FU. The mechanism of GC cell resistance to 5-FU is currently unknown.

**Materials and Methods:**

This study employed next-generation sequencing (NGS) to analyze the differentially expressed genes (DEGs) in chemotherapy-sensitive and non-sensitive GC tissues. In addition, a bioinformatics analysis was conducted using the GC dataset of GEO, and further validated and explored through *in vitro* experiments.

**Results:**

Thyroid adenoma-associated gene (THADA) was highly expressed in GC tissues from chemotherapy-sensitive patients and was an independent prognostic factor in GC patients receiving postoperative 5-FU adjuvant chemotherapy. Notably, heightened THADA expression in GC cells was associated with the down-regulation of autophagy-related proteins (LC-3, ATG13, ULK1, and TFEB). Furthermore, the PI3K/AKT/mTOR signaling pathway and mTORC1 signaling pathway were remarkably increased in patients with elevated THADA expression. THADA expression was associated with mTOR, the core protein of the mTOR signaling pathway, and related proteins involved in regulating the mTORC1 signaling pathway (mLST8, RHEB, and TSC2). THADA exhibited inhibitory effects on autophagy and augmented the sensitivity of GC cells to 5-FU through the PI3K/AKT/mTOR signaling pathway.

**Conclusion:**

The findings suggest that THADA may be involved in the regulatory mechanism of GC cell sensitivity to 5-FU. Consequently, the detection of THADA in tumor tissues may bring clinical benefits, specifically for 5-FU-related chemotherapy administered to GC patients with elevated THADA expression.

## Introduction

Gastric cancer (GC) is a highly prevalent gastrointestinal malignancy worldwide, characterized by high recurrence and mortality rates. The overall 5-year survival rate for GC is less than 30% (1). Due to the lack of effective targeted drugs, chemotherapy remains the main clinical treatment for GC adjuvant and systemic therapy (2). Among the chemotherapy regimens, 5-Fluorouracil (5-FU) is currently the primary drug in the mainstream chemotherapy regimen for GC (3). Over the years, administrative strategies of 5-FU in GC have been optimized, including the incorporation of sensitizer folinic acid and the development of oral dosage formulations, such as esvam (S-1) and capecitabine (4, 5). However, insensitivity and acquired resistance are considered the primary clinical challenges in 5-FU therapy (6). The mechanism responsible for GC’s resistance to 5-FU is currently unknown.

Autophagy, a physiological cellular process involved in the degradation of damaged proteins and organelles, plays a crucial role in self-metabolism, apoptosis, growth, and the maintenance of intracellular homeostasis (7). It is well recognized that autophagy may play two different pivotal roles, either by promoting or suppressing tumor development. Moreover, conclusive findings from previous studies have demonstrated that autophagy induced by antitumor drugs in cancer cells may lead to chemotherapy resistance (8). Several studies have demonstrated that autophagy confers protection to cancer cells from apoptosis mediated by DNA-damaging drugs (e.g., 5-FU) through diverse mechanisms, including induction of cellular senescence, delayed division, and cell cycle arrest (9, 10).

Alterations in chemotherapeutic drug-related signaling pathways are important potential mechanisms of 5-FU resistance, especially those that play important roles in tumor cell growth and proliferation (11, 12). These signaling pathways are responsive to relevant intra- and extracellular factors in addition to the cellular environment and subsequently are activated to modulate cellular functions. Notably, It has been indicated that PI3K/AKT/mTOR signaling pathways may be regulated by diverse extrinsic factors, which ultimately affect the sensitivity of tumor cells to 5-FU (13).

THADA, a gene associated with cold adaptation in humans, plays a pivotal role in energy homeostasis (14). Furthermore, THADA has been primarily involved in the regulation of body metabolism and implicated in certain diseases including diabetes and polycystic ovary syndrome (15-17). However, the precise functional implications of THADA in tumorigeneses are relatively unexplored. Notably, studies have demonstrated that chromosomal aberrations of THADA are associated with benign thyroid adenomas (18). Although several studies have reported that THADA is associated with increased risk of multiple malignancies, the function and molecular mechanisms of THADA in malignancies remain unclear (19, 20). Certain THADA fusion-positive cancers, tend to exhibit a lower grade and more benign characters (21). However, in certain aggressive poorly differentiated thyroid cancers, aberrant THADA fusions promote increased cell proliferation, migration, and transformation (20). Additionally, a study identified that THADA is a key regulator in maintaining PD-L1 expression in colon cancer, with THADA expression in colon cancer tissues exhibiting a positive correlation with PD-L1 expression. PD-L1 could be specifically inhibited by silencing THADA, which promotes antitumor effects by enhancing T cell cytotoxicity (22). This study reveals the first exploration of the effect of THADA on the sensitivity of 5-FU chemotherapy in GC patients and its clinical significance.

## Materials and Methods

***Patients and samples***

In this study, tumor tissue from initial gastroscopic biopsies of 9 GC patients who received neoadjuvant chemotherapy (NAC), was collected for next-generation sequencing (NGS) as displayed in the Supplementary Table. All patients underwent imaging re-assessment and radical gastrectomy after 2 cycles of NAC (SOX: S-1 and oxaliplatin). 

***Assessment of chemotherapy efficacy***

The evaluation of chemotherapy efficacy based on the imaging assessment was classified according to RECIST 1.1 criteria: Partial Response (PR), Stable Disease (SD), and Progressive Disease (PD). Certain patients with postoperative pathology indicating no invasive cancer cells remaining in the primary site and lymph nodes were considered to be pathologic complete response (pCR). Meanwhile, patients achieving pCR and PR were considered chemotherapy-sensitive. Nevertheless, other patients achieving SD and PD were considered chemotherapy-insensitive (Supplementary Table).

***Analysis of differentially expressed genes (DEGs)***

In this study, the GC microarray dataset (GSE31811) data of 19 patients with metastatic GC who received DCS combination chemotherapy regimens (S-1, cisplatin, and docetaxel) were obtained from the GEO database, including 11 chemotherapy-sensitive and 8 chemotherapy-insensitive samples. 

The DEGs between chemotherapy-sensitive and non-sensitive samples in our NGS data and dataset GSE31811 were analyzed using the limma package of R software, and the expression foldchange (FC) of each RNA control sample and experimental group samples was calculated and logarithmic values were taken to obtain log2FC. Based on adj. *P*-value<0.05 and log2 FC absolute value >2 to screen for genes with significant differential expression.

The heatmap.2 package of R software was used to plot the DEGs heatmap of the data. The ggplot2 package of R software was used to plot the DEGs volcano of the data. 

***Survival analysis of GEO data***

In this study, data from the GC microarray datasets (GSE66229 and GSE26253) were obtained from the GEO database for survival analysis. The dataset GSE66229 includes gene expression data and survival data from 300 GC patients who underwent surgery alone. In contrast, the dataset GSE26253 includes gene expression data and survival data from 432 GC patients who underwent both surgery and postoperative 5-FU adjuvant chemotherapy. Postoperative pathological staging was conducted according to the 8th edition of the AJCC/UICC TNM staging of GC.

***GSEA analysis*** 

In this study, GSEA analysis was performed using the GEO dataset (GSE62254, 300 GC samples) and divided GC patients into high (n=150) and low (n=150) expression groups according to THADA expression, and the augmentation of tumor-related signaling pathways were analyzed between the two groups (Geneset database: C6-oncogenic gene sets). The false discovery rate (FDR) in GSEA was obtained after multiple calculations according to the built-in program, and FDR <0.25 and nominal *P*-value<0.05 were considered statistically different.

***Statistical analysis***

Statistical analyses were conducted using the software SPSS version 19.0 and GraphPad Prism version 9.02. Continuous variables were tested by t-test and nonparametric test. Overall survival (OS) was calculated for survival analysis, with survival time being the time from surgery to death or the last follow-up. The Kaplan-Meier method and log-rank test were employed for univariate survival analysis, and the Cox proportional risk model was utilized for multivariate analysis. *P*<0.05 was considered to be statistically significant.

## Results

**Figure 1:**
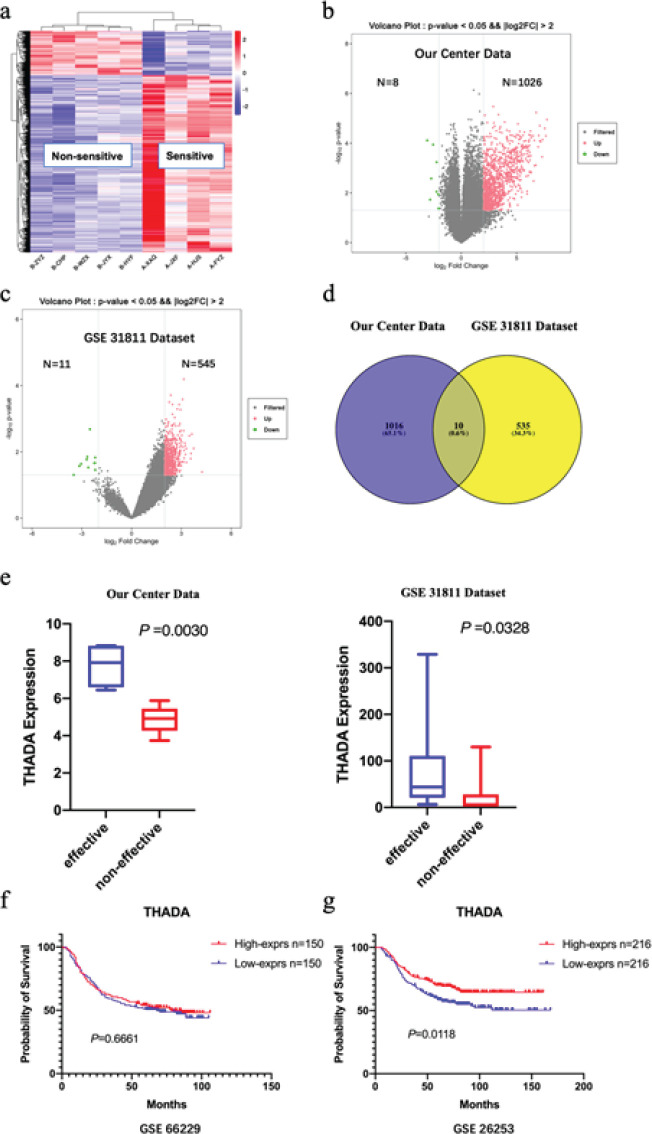
Differentially expressed genes (DEGs) in chemotherapy-sensitive gastric cancer (GC) samples and the survival significance of thyroid adenoma-associated gene (THADA)

In this study, THADA is highly expressed in GC tissues of chemotherapy-sensitive patients. 1026 up-regulated DGEs and 8 down-regulated DGEs were discovered in the chemotherapy-sensitive samples (Figure 1a, b). Meanwhile, analysis of DEGs utilizing the GC microarray dataset GSE31811 on GEO revealed 545 up-regulated DGEs and 11 down-regulated DGEs in chemotherapy-sensitive samples (Figure 1c). In further analysis, a total of 10 common DEGs were up-regulated in GC tissues of chemotherapy-sensitive samples in both our data and dataset GSE31811 (Figure 1d), which included THADA. Low THADA expression is an independent poor prognostic factor in GC patients receiving postoperative 5-FU adjuvant chemotherapy.

THADA expression was significantly up-regulated in the chemotherapy-sensitive group of our center data and GEO dataset GSE31811 (*P*=0.003 and 0.0328, respectively; Figure 1e). A univariate survival analysis using the GEO dataset GSE66229 (300 patients) revealed no survival difference between the THADA high and low expression groups in GC patients who underwent surgery alone without adjuvant chemotherapy (*P*=0.6661; Figure 1f). However, patients who underwent both surgery and postoperative 5-FU adjuvant chemotherapy (GEO dataset GSE26253, 432 patients), and those with low THADA expression displayed shorter OS (*P*=0.0118; Figure 1g). After including TNM stage and THADA expression together in a multifactorial survival analysis, both high TNM stage (*P*=0.008) and low THADA expression (*P*=0.038) were found to be independent poor prognostic factors for GC patients receiving postoperative 5-FU adjuvant chemotherapy (Table 1).

***THADA enhances the sensitivity of GC cells to 5-FU***

**Figure 2:**
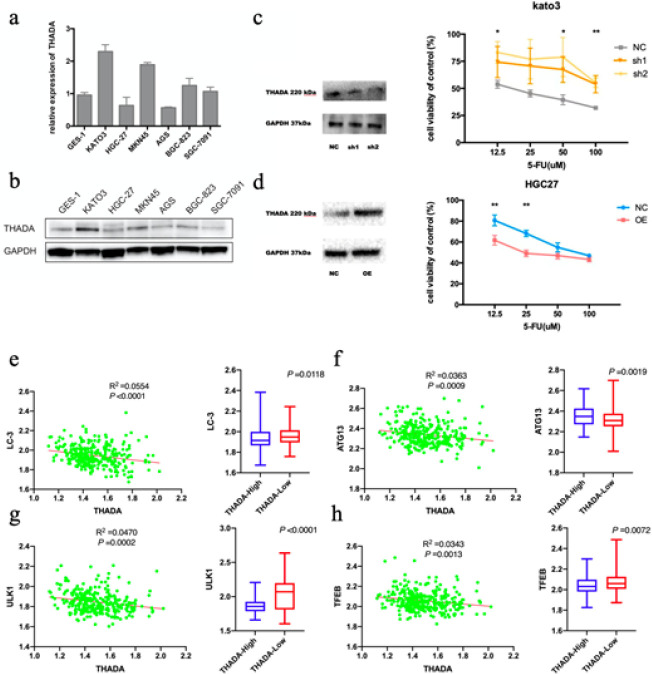
Thyroid adenoma-associated gene (THADA) affects the sensitivity of gastric cancer (GC) cells to 5-Fluorouracil (5-FU) and is associated with down-regulation of autophagy

The expressions of THADA varied in human gastric mucosal cell GES-1 and GC cell lines. THADA expression was elevated in KATO3 cells and lower in HGC-27 cells (Figure 2a, b). In this study, THADA expression in KATO3 GC cells was knocked down by transfection of shRNA plasmid directed against THADA. The 5-FU IC50 concentration of NC KATO3 GC cells was 17.29 μM. Meanwhile, that of THADA sh1 KATO3 GC cells was 179.2 μM, and that of THADA sh2 KATO3 GC cells was 175.6 μM. The sensitivity of KATO3 GC cells to 5-FU was reduced after the knockdown of THADA (Figure 2c). For the overexpression, the coding DNA sequence of THADA was cloned into pReceiver-Lv201 plasmid and then transfected into HGC-27 GC cells. The 5-FU IC50 concentration of NC HGC-27 GC cells was 75.71 μM, while that of THADA OE HGC-27 GC cells was 36.51 μM. The sensitivity of HGC-27 GC cells to 5-FU was increased after overexpression of THADA (Figure 2d).

***High THADA expression in GC cells is associated with down-regulation of autophagy***

In this study, using the GEO dataset GSE62254 (300 patients) for analysis, GC patients were divided into two different groups in accordance with high and low THADA expression. The expression of autophagy microtubule-associated protein light chain 3 (LC-3), autophagy-related protein 13 (ATG13), unc51-like autophagy activating kinase 1 (ULK1), and the transcription factor EB (TFEB) in GC tissues was analyzed between the two groups. The expression of LC-3, ATG13, ULK1, and TFEB was detected to be lower in GC tissues of patients with high THADA expression (*P*=0.0118, 0.0019, <0.0001, and 0.0072, respectively) and negatively correlated with THADA expression (R^2^=0.0554, 0.0363, 0.0470, and 0.0343, respectively; *P*<0.0001, 0.0009, 0.0002, and 0.0013, respectively; Figure 2e, f, g, h).

***High THADA expression in GC cells is associated with augmentation of mTOR complex 1 (mTORC1) signaling pathway***

**Figure 3:**
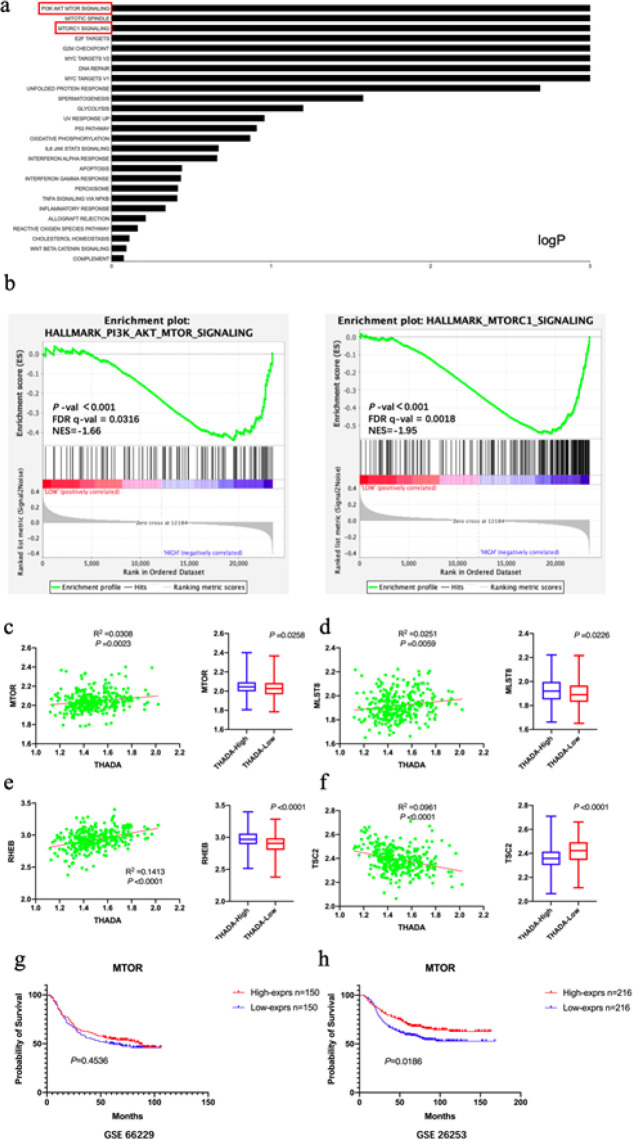
Relationship between thyroid adenoma-associated gene (THADA) expression and mammalian target of rapamycin (mTOR) signaling pathway in gastric cancer (GC) tissues and the survival significance of mTOR expression

In this study, GC patients in the GEO dataset GSE62254 were divided into two groups according to THADA expression (150 High vs 150 Low), and the augmentation of tumor-associated signaling pathways between the two groups was analyzed by using GSEA. The mTORC1 signaling pathway (*P*<0.001, NES=-1.95) and PI3K/AKT/mTOR signaling pathway (*P*<0.001, NES=-1.66; Figure 3a, b) were found to be highly enriched in GC tissues from patients with high THADA expression.

***mTORC1 signaling pathway-related proteins correlated with THADA expression*** 

The expression of the mammalian target of rapamycin (mTOR), and the core protein of the mTOR signaling pathway, were positively correlated with THADA (R^2^=0.0308, *P*=0.0023), and were elevated in GC tissues with high THADA expression (*P*=0.0258; Figure 3c). Meanwhile, the relationship between THADA and main related proteins involved in regulating the mTORC1 signaling pathway (mammalian lethal with SEC13 protein 8, mLST8; ras homolog enriched in brain, RHEB; tuberous sclerosis complex 2, TSC2) were further analyzed. The mLST8 and RHEB expression was elevated in GC tissues from patients with high THADA expression (*P*=0.0226 and <0.0001, respectively) and positively correlated with THADA (R^2^=0.0251 and 0.1413, respectively; *P*=0.0059 and <0.0001, respectively; Figure 3de). In contrast, the TSC2 expression was lower in GC tissues of patients with high THADA expression (*P*<0.0001) and negatively correlated with THADA (R^2^=0.0961, *P*<0.0001; Figure 3f). 

***Low mTOR expression is an independent poor prognostic factor in GC patients receiving postoperative 5-FU adjuvant chemotherapy***

A univariate survival analysis utilizing the GEO dataset revealed no survival difference between the mTOR high and low expression groups in GC patients who received surgery alone without adjuvant chemotherapy (GSE66229, 300 patients) (*P*=0.4536; Figure 3g). However, patients who underwent both surgery and postoperative 5-FU adjuvant chemotherapy (GEO dataset GSE26253, 432 patients), in addition to patients with low mTOR expression had reduced OS (*P*=0.0186; Figure 3h). After including the TNM stage, THADA expression, and mTOR expression collectively in a multifactorial survival analysis, it was observed that the advanced TNM stage (*POS*<0.001), low THADA expression (*P*=0.048), and low mTOR (*P*=0.008) were found to be independent poor prognostic factors for GC patients receiving postoperative 5-FU adjuvant chemotherapy (Table 2).

***THADA inhibits autophagy in GC cells through the PI3K/Akt/mTOR signaling pathway and increases the sensitivity of GC cells to 5-FU***

**Figure 4:**
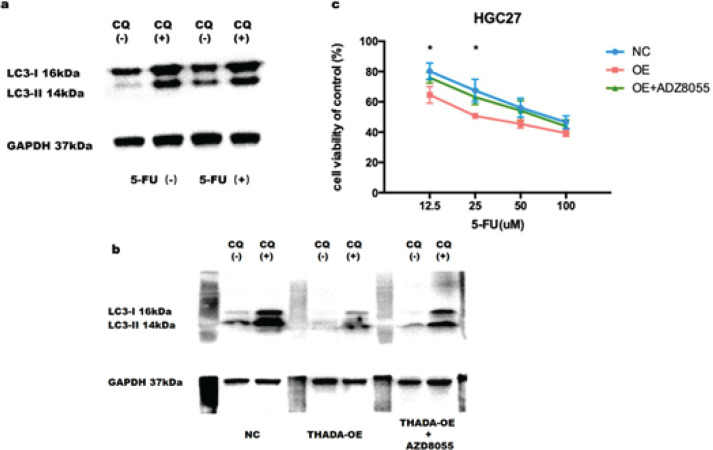
Thyroid adenoma-associated gene (THADA) affects gastric cancer (GC) cell autophagy and sensitivity to 5-Fluorouracil (5-FU) via the PI3K/Akt/mTOR signaling pathway

The expression of LC3 II in KATO3 GC cells was increased in the presence of 5-FU, and the accumulation of LC3II was further elevated in the presence of chloroquine, suggesting elevated autophagy (Figure 4a). Meanwhile, in THADA-OE HGC27 cells, LC3-II expression was down-regulated and accumulation of LC3-II was attenuated after treatment with chloroquine, suggesting that autophagy was inhibited after THADA overexpression in GC cells. In contrast, the expression of LC3 II increased again in THADA-OE HGC27 cells after treatment with mTOR kinase inhibitor AZD8055, and the accumulation of LC3II further increased under the effect of chloroquine, indicating that the inhibition of autophagy caused by the overexpression of THADA was reversed after inhibition of the PI3K/Akt/mTOR signaling pathway (Figure 4b). 

In the presence of 5-FU, the 5-FU IC50 concentration in NC HGC-27 GC cells was 76.37 μM, whereas the 5-FU IC50 concentration in THADA OE HGC-27 GC cells was 35.24 μM, suggesting that the sensitivity of GC cells to 5-FU was increased after THADA overexpression in GC cells. In contrast, the 5-FU IC50 concentration in THADA-OE HGC27 GC cells increased again to 65.55 uM after treatment with mTOR kinase inhibitor AZD8055, suggesting that the sensitivity of GC cells to 5-FU decreased once more after inhibition of PI3K/Akt/mTOR signaling pathway (Figure 4c).

a) Heat map of gene expression in chemotherapy-sensitive (5 cases) and non-sensitive (4 cases) GC tissue samples from our center data. Expression is up-regulated in red and down-regulated in blue. b) Volcano plot of DEGs from our center data, with 1026 up-regulated DEGs and 8 down-regulated DEGs in the chemotherapy-sensitive group. c) Volcano plot of DEGs from GEO dataset GSE31811, with 545 up-regulated DEGs and 11 down-regulated DEGs in the chemotherapy-sensitive group. d) Up-regulated DEGs in the Venny diagram. The common up-regulated DEGs are: THADA; GNAS; NDUFAF6; WWOX; PDXDC2P; ATF7IP; LUC7L3; CCT7; UBD; MED10. e) THADA expression was higher in chemotherapy-sensitive GC patients in our data and the GEO dataset GSE31811. f) Survival differences in 300 GC patients who received surgery alone without adjuvant chemotherapy (GSE66229) with different THADA expression. g) Survival differences in 432 GC patients who received surgery and 5-FU adjuvant chemotherapy (GSE26253) with different THADA expression

**Table 1:** Cox multivariate analysis of THADA

Factors	Cox multivariate analysis^c^	
	*P*-Value	HR (95% CI)^d^
TNM Stage (I, II, III, IV) ^a^	0.008	0.600 (0.411, 0.875)
THADA Expression (High vs. Low)^b^	0.038	1.387 (1.018, 1.891)

^a ^Postoperative pathological staging was performed according to the 8th edition of the AJCC/UICC TNM staging of gastric cancer. TNM Stage I: 68 cases; TNM Stage II: 167 cases; TNM Stage III: 130 cases; TNM Stage IV: 67 cases; ^b ^THADA Expression High: 216 cases; THADA Expression Low: 216 cases; ^c ^Forward likelihood ratio method

dHR: Hazard Ratio; CI: Confidence Interval

a, b) THADA expression in human gastric mucosal cells (GES-1) and GC cell lines (KATO3, HGC-27, MKN-45, AGS, BGC-823, SGC-7901) were detected by western blot and Q-PCR, respectively. c) Expression of THADA was knocked down in KATO3 GC cells by transfection with shRNA knockdown THADA lentiviral plasmid. NC: NC KATO3 GC cells; sh1: THADA sh1 KATO3 GC cells; sh2: THADA sh2 KATO3 GC cells. The cell survival rate under different concentrations of 5-FU treatment was detected using cell counting kit-8 (CCK-8). d) THADA expression was overexpressed in HGC-27 GC cells by transfection with a THADA overexpressing lentiviral plasmid. NC: NC HGC-27 GC cells; OE: THADA-OE HGC27 GC cells. The cell survival rate under different concentrations of 5-FU treatment was detected using cell counting kit-8 (CCK-8). The expression of LC-3 (e), ATG13 (f), ULK1 (g), and TFEB (h) was lower in THADA high-expressing GC tissues and negatively correlated with the expression of THADA. Based on the GEO dataset GSE62254

a) High enrichment of tumor-associated signaling pathways in THADA high expressing GC tissues. Enrichment score (ES) significance *P-*value was chosen as the logarithm (-log10) of the horizontal coordinate. Signaling pathways with a *P-*value less than 0.001 were taken as *P-*value=0.001. b) Enrichment analysis plots of PI3K/AKT/mTOR signaling pathway and mTORC1 signaling pathway. c, d, e) Expression of mTOR, mLST8, and RHEB was up-regulated in THADA high-expressing GC tissues and positively correlated with THADA expression. f) Expression of TSC2 was down-regulated in THADA high-expressing GC tissues and negatively correlated with THADA expression. Based on the GEO dataset GSE62254. g) Survival differences between the two groups of high and low mTOR expression in 300 GC patients who received surgery alone without adjuvant chemotherapy (GSE66229). h) Survival differences between the two groups of high and low mTOR expression in 432 GC patients who received surgery alone without adjuvant chemotherapy (GSE26253)

**Table 2:** Cox multivariate analysis of mammalian target of rapamycin (mTOR)

Factors	Cox multivariate analysis^d^	
	*P*-Value	HR (95% CI)^e^
TNM Stage (I, II, III, IV)^a^	<0.001	2.054 (1.733, 2.435)
THADA Expression (High vs. Low)^b^	0.048	0.733 (0.539, 0.998)
mTOR Expression (High vs. Low)^c^	0.008	1.518 (1.117, 2.064)

^a^ Postoperative pathological staging was performed according to the 8th edition of the AJCC/UICC TNM staging of gastric cancer. TNM Stage I: 68 cases; TNM Stage II: 167 cases; TNM Stage III: 130 cases; TNM Stage IV: 67 cases; ^b^THADA Expression High: 216 cases; THADA Expression Low: 216 cases; ^c ^mTOR Expression High: 216 cases; mTOR Expression Low: 216 cases; ^d ^Forward likelihood ratio method

eHR: Hazard Ratio; CI: Confidence Interval

a) Autophagy was up-regulated in GC cells after 5-FU treatment. Cells were treated with 12.5 μM of 5-FU for 48 hr in the experimental group. b) THADA down-regulates autophagy in GC cells via the PI3K/Akt/mTOR signaling pathway. c) THADA enhances the sensitivity of GC cells to 5-FU via the PI3K/Akt/mTOR signaling pathway. NC: NC HGC-27 GC cells. OE: THADA-OE HGC27 GC cells; OE+ADZ8055: THADA-OE HGC27 GC cells were treated with mTOR kinase inhibitor AZD8055 for 72 hr; CQ: Chloroquine 50 μM; The cell survival rate under different concentrations of 5-FU treatment was detected using cell counting kit-8 (CCK-8)

PI3K: Phosphoinositide 3-kinase; AKT: PKB, protein kinase B; mTOR: Mammalian target of rapamycin

**Figure 5:**
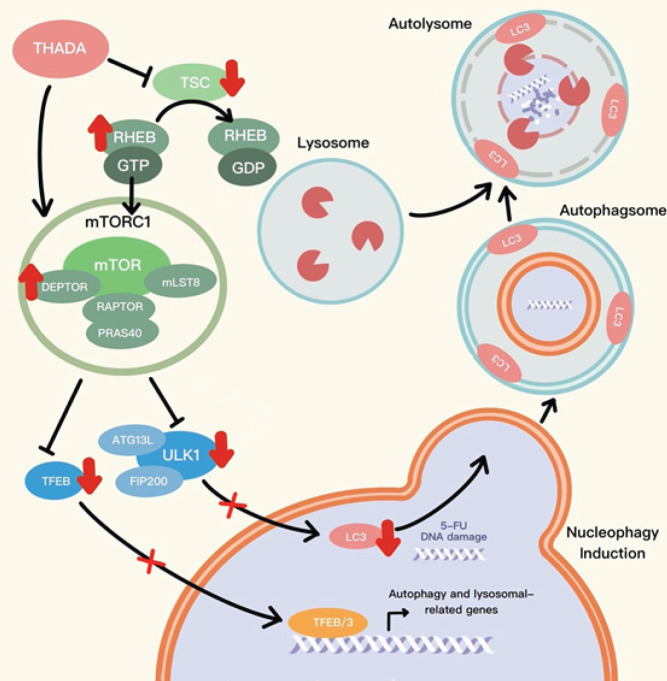
Hypotheses: up-regulation of Thyroid adenoma-associated gene (THADA) expression in gastric cancer (GC) cells inhibits autophagy through the PI3K/AKT/mTOR signaling pathway, especially the mTORC1 signaling pathway, which in turn enhances the sensitivity of GC cells to 5-Fluorouracil (5-FU)

PI3K: Phosphoinositide 3-kinase; AKT: PKB, protein kinase B; mTOR: Mammalian target of rapamycin

## Discussion

In recent years, the application of NGS in gene expression has become increasingly valuable in clinical studies including molecular classification, prognosis prediction, and new target discovery (23, 24). In the present study, we found that THADA was remarkably expressed in tumor tissues of chemotherapy-sensitive GC patients by NGS of clinical GC samples and bioinformatics analysis of GEO data, whereas decreased THADA expression in tumor tissues was found to be an independent poor prognostic factor in GC patients receiving postoperative 5-FU adjuvant chemotherapy. Nevertheless, further *in vitro* experiments confirmed that THADA expression in GC cells could enhance the sensitivity of GC cells to 5-FU.

In advanced tumor stages, autophagy becomes a mechanism of cellular self-protection for maintaining mitochondrial function, reducing DNA damage in addition to enhancing survival under stressful environments (e.g., DNA damage metabolic stress and chemotherapy) and ultimately causing resistance to antitumor drugs and promoting tumor progression (25, 26). Studies have shown that 5-FU treatment induces autophagy within tumor cells. However, at present, inhibition of autophagy enhances the anticancer effect of 5-FU (27-29). The protective effect of autophagy against cytotoxicity during 5-FU treatment of GC has been demonstrated (30, 31). *In vitro* and *in vivo* experiments have demonstrated that long noncoding RNA (lncRNA) EIF3J-DT can activate autophagy in GC cells and further induce 5-FU resistance by targeting autophagy-related protein 14 (ATG14) in the presence of 5-FU (32). The cyclic RNA counts per million (CPM) can promote 5-FU chemoresistance in GC cells by activating protein kinase AMP‐activated catalytic subunit alpha 2 (PRKAA2) -mediated autophagy (33). Inhibition of GC cell autophagy by the small interfering RNA (siRNA) of class III PI3K can promote the antitumor effects of 5-FU (34). In the present study, 5-FU was observed to induce the up-regulation of autophagy in GC cells, an observation that is consistent with previous findings.

The various steps of autophagy are mainly negative-regulated by the mTORC1 signaling pathway (35). In this process, ATG13 and ULK1 are the key substrates in the regulation of autophagy by mTORC1. In addition, ULK1 and ATG13 can form the ULK1/ ATG13/FIP200 complex which promotes early steps of autophagy. Concurrently, mTORC1 can directly inhibit autophagy by down-regulating the formation of this complex (36). In addition, mTORC1 can enhance the early blockade of autophagy by inhibiting TFEB and its family members (37). In this study, we found that the expression of autophagy marker LC-3 and autophagy-related facilitators (ULK1, ATG13, and TFEB) was decreased in GC tissues from patients with elevated THADA expression and negatively correlated with THADA, suggesting that THADA in GC cells may be associated with negative regulation of autophagy associated with the mTORC1 signaling pathway.

The rapid proliferation of cancer cells requires large amounts of energy and macromolecules. In addition, it is often accompanied by metabolic abnormalities (38). Therefore, alterations in the PI3K/AKT/mTOR signaling pathway are common in malignancies. The PI3K/AKT/mTOR signaling pathway acts mainly through mTORC1 and mTOR complex 2 (mTORC2). Previous studies have shown that mTORC1 is dysregulated in a variety of cancer types, including breast, cervical, esophageal squamous cell, lung, and liver cancers (39-43). Among numerous *in vitro* cell lines and *in vivo* mouse xenograft models, abnormal activation of mTORC1 contributes to tumor growth, angiogenesis, invasion, and metastasis (44). mTORC1 plays a key regulatory role in processes related to cancer cell growth and metabolism. In this study, we found that the mTORC1 signaling pathway and the PI3K/AKT/mTOR signaling pathway were highly augmented in the THADA high expression group using GSEA analysis.

The mTORC1 signaling pathway is usually activated by its upstream regulators (45). Among the many regulators of mTORC1, mLST8, which binds to the catalytic structural domain of mTOR and stabilizes kinase activation, is an important component of mTORC1 and positively regulates the mTORC1 signaling pathway (46). In addition, the mTORC1 signaling pathway is positively regulated by RHEB. When bound to GTP, RHEB induces conformational changes in mTORC1, leading to kinase activation (47). Meanwhile, TSC2, a GTPase-activating protein of RHEB, negatively regulates mTORC1 by promoting RHEB-GTP hydrolysis and converting RHEB to an inactive GDP-bound state (48). In this study, we found elevated mTOR expression in GC tissues of patients with high THADA expression. Meanwhile, the expression of mLST8 and RHEB, up-regulators of the mTORC1 signaling pathway, was elevated in GC tissues from patients with an increased THADA expression; while the expression of TSC2, a down-regulator of the mTORC1 signaling pathway, was lower. This suggests that THADA in GC cells may be related to the activation of the PI3K/AKT/mTOR signaling pathway, especially the mTORC1 signaling pathway. In addition, we found that the sensitivity of GC cells to 5-FU was up-regulated after overexpression of THADA in GC cells. Meanwhile, autophagy in GC cells was inhibited. After inhibiting the PI3K/AKT/mTOR signaling pathway in GC cells by AZD8055, the alteration of autophagy and 5-FU sensitivity caused by THADA overexpression could be reversed.

The findings presented above suggest that THADA may have an inhibitory role in 5-FU-induced protective autophagy by up-regulating the activation of PI3K/AKT/mTOR signaling pathway in GC cells, particularly the mTORC1 signaling pathway, and thus increase the sensitivity of GC cells to 5-FU. However, the specific mechanism is required to be elucidated by further clinical sample data and validated through additional experiments.

## Conclusion

In this study, we found a potential link between THADA and chemotherapy sensitivity. All results suggest that the up-regulation of THADA expression in GC cells inhibits autophagy through the PI3K/AKT/mTOR signaling pathway, especially the mTORC1 signaling pathway, which in turn enhances the sensitivity of GC cells to 5-FU. The hypotheses derived from these findings necessitate further investigation to validate and expand upon the observed results (Figure 5). Meanwhile, as a potential marker of chemotherapy sensitivity, THADA detection may bring clinical benefits of 5-FU-related chemotherapy for GC patients with high THADA expression.

## Authors’ Contributions

J Y and X L designed the experiments; X L and J P performed experiments; H H collected data; X L discussed the results and strategy; J Y supervised, directed, and managed the study; J Y, X L, and J P finally approved the version to be published.

## Funding

None.

## Conflicts of Interest

The authors indicated no potential conflicts of interest.
